# Percutaneous Versus Surgical Femoral Cannulation in Minimally Invasive Cardiac Surgery: A Systematic Review and Meta-Analysis

**DOI:** 10.1177/15569845241241534

**Published:** 2024-04-11

**Authors:** Hristo Kirov, Tulio Caldonazo, Angelique Runkel, Johannes Fischer, Panagiotis Tasoudis, Murat Mukharyamov, Gianmarco Cancelli, Michele Dell’Aquila, Torsten Doenst

**Affiliations:** 1 Department of Cardiothoracic Surgery, Friedrich-Schiller-University Jena, Germany; 2 Division of Cardiothoracic Surgery, University of North Carolina at Chapel Hill, NC, USA; 3 Department of Cardiothoracic Surgery, Weill Cornell Medicine, New York, NY, USA

**Keywords:** minimally invasive cardiac surgery, femoral cannulation, percutaneous cannulation

## Abstract

**Objective:**

Minimally invasive cardiac surgery (MICS) is increasing worldwide. In most cases, the surgical technique includes cannulation of the groin for the establishment of cardiopulmonary bypass, requiring a second surgical incision (SC) for exposure and cannulation of the femoral vessels. With the introduction of arterial closure devices, percutaneous cannulation (PC) of the groin has become a possible alternative. We performed a meta-analysis and systematic review to compare clinical endpoints between the patients who underwent PC and SC for MICS.

**Methods:**

Three databases were assessed. The primary outcome was any access site complication. Secondary outcomes were perioperative mortality, any wound complication, any vascular complication, lymphatic complications, femoral/iliac stenosis, stroke, procedural duration, and hospital length of stay (LOS). A random effects model was performed.

**Results:**

A total of 5 studies with 2,038 patients were included. When compared with PC, patients who underwent SC showed a higher incidence of any access site complication (odds ratio [OR] = 3.09, 95% confidence interval [CI]: 1.87 to 5.10, *P* < 0.01), any wound complication (OR = 10.10, 95% CI: 3.31 to 30.85, *P* < 0.01), lymphatic complication (OR = 9.37, 95% CI: 2.15 to 40.81, *P* < 0.01), and longer procedural duration (standardized mean difference = 0.31, 95% CI: 0.12 to 0.51, *P* < 0.01). There was no significant difference between the 2 groups regarding perioperative mortality, any vascular complication, femoral/iliac stenosis, stroke, or hospital LOS.

**Conclusions:**

The analysis suggests that surgical groin cannulation in MICS is associated with a higher incidence of any access site complication (especially wound complication and lymphatic fistula) and with a longer procedural time compared with PC. There was no difference in perioperative mortality.

## Graphical abstract

**Visual abstract:**
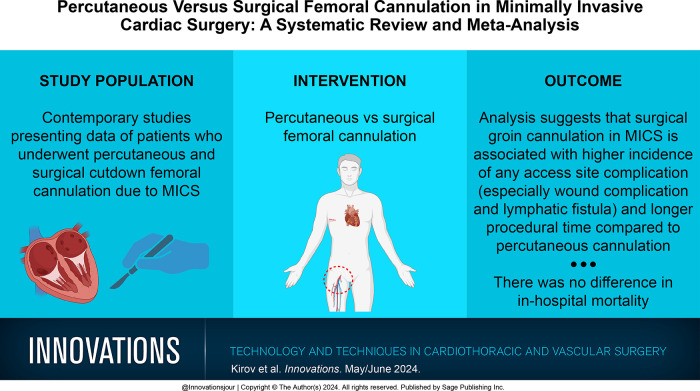

**Central Message**

Surgical groin cannulation in MICS carries a higher risk of access site complication and longer procedure time, but it does not affect perioperative mortality. Centers specializing in minimally invasive procedures might consider adopting percutaneous groin cannulation to minimize perioperative wound complications and enhance patient safety.

## Introduction

Minimally invasive cardiac surgery (MICS) has become an established alternative to classic sternotomy approaches, with increasing numbers of procedures performed worldwide.^1^ With survival being practically the same between sternotomy and MICS approaches, secondary endpoints and complication rates, including complication rates after cannulation for cardiopulmonary bypass (CPB), have moved center stage.^1^ In most cases, the MICS surgical technique includes not only a thoracotomy but also cannulation of the groin for the establishment of CPB, requiring a second surgical incision for exposure and cannulation of the femoral vessels. In the past decade, with the introduction of several arterial closure devices (ACDs) and their regular use in the field of transcatheter aortic valve replacement, percutaneous cannulation of the groin for MICS has become a possible alternative. However, except for the obvious cosmetic benefit of the percutaneous approach, the different technique of cannulation and arterial closure with ACDs in percutaneous cannulation may give rise to different types and rates of complications.

We have already reported our experience with percutaneous cannulation, showing that percutaneous cannulation might reduce complication rates, but if complications arise they are mainly vascular in nature.^2^ Several other centers have reported their outcomes, which are partly similar but may also differ from ours. So far, a systemic evaluation for the comparison of complication rates in surgical or percutaneous cannulation groin cannulation is missing. Therefore, we performed a meta-analysis and systematic review to compare all relevant clinical endpoints between patients who underwent percutaneous and surgical cannulation for MICS.

## Methods

Ethical approval of this analysis was not required as no human or animal subjects were involved. This review was registered with the National Institute for Health Research International Registry of Systematic Reviews (PROSPERO, CRD42023431936).

### Search Strategy

We performed a comprehensive literature search to identify contemporary studies reporting short-term outcomes between patients who underwent percutaneous and surgical cutdown femoral cannulation due to MICS. Searches were run in June 2023 in the following databases: Ovid MEDLINE, ScienceDirect, and The Cochrane Library (Wiley). The search strategy for Ovid MEDLINE is available in Supplemental Table 1.

### Study Selection and Data Extraction

The study selection followed the Preferred Reporting Items for Systematic Reviews and Meta-Analyses (PRISMA) strategy. After de-duplication, records were screened by 2 independent reviewers. Any discrepancies and disagreements were resolved by a third author. Titles and abstracts were reviewed against predefined inclusion and exclusion criteria. Studies were considered for inclusion if they were written in English and reported a direct comparison between patients who underwent percutaneous and surgical cutdown femoral cannulation due to MICS. Animal studies, abstracts, case reports, commentaries, editorials, expert opinions, conference presentations, review articles, studies published before 2013, and studies not reporting the outcomes of interest were excluded. The full text was pulled for the selected studies for a second round of eligibility screening. References for selected articles were also reviewed for relevant studies not captured by the original search. Risk of bias was assessed based on Newcastle-Ottawa assessment scale (Supplemental Table 2).

Two reviewers independently performed data extraction. Accuracy was verified by a third author. The extracted variables included study characteristics (publication year, country, sample size, study design, enrollment dates, presence or absence from population adjustment, and reported outcomes) as well as patient demographics (age, sex, body mass index, hypertension, diabetes, peripheral vascular disease, atrial fibrillation, and prior cardiac surgery). Supplemental Table 3 shows the surgical techniques and devices used in the individual studies.

### Outcomes

The primary outcome was any access site complication. Secondary outcomes were perioperative mortality (defined as in-hospital or 30-day mortality), any wound complication, any vascular complication, lymphatic complications, femoral/iliac stenosis, stroke, procedural duration, and hospital length of stay.

### Statistical Analysis

We conducted meta-analyses to compare the outcomes of percutaneous and surgical cutdown femoral cannulation. Odds ratios (ORs) and 95% confidence intervals (CIs) were calculated for each outcome. An OR greater than 1 indicated that the outcome was more frequently present in the surgical cannulation arm. Continuous variables were analyzed using standardized mean difference (SMD) and 95% CI. An SMD greater than zero corresponded to larger values in the percutaneous cannulation arm. Inherent clinical heterogeneity between the studies was balanced via the implementation of random effects models (DerSimonian–Laird). Results were displayed in forest plots. Alpha error was set to 0.05.

Between-study statistical heterogeneity was assessed with the Cochran Q statistic and by estimating I^2^. High heterogeneity was confirmed with a significance level of *P* < 0.10 and I^2^ of at least 50% or more. Leave-one-out sensitivity analyses were also performed for the primary outcome. All statistical analyses were performed using R version 4.1.1 (The R Foundation, Vienna, Austria) within RStudio (Posit, Boston, MA, USA).

## Results

### Study Characteristics

**Fig. 1.**
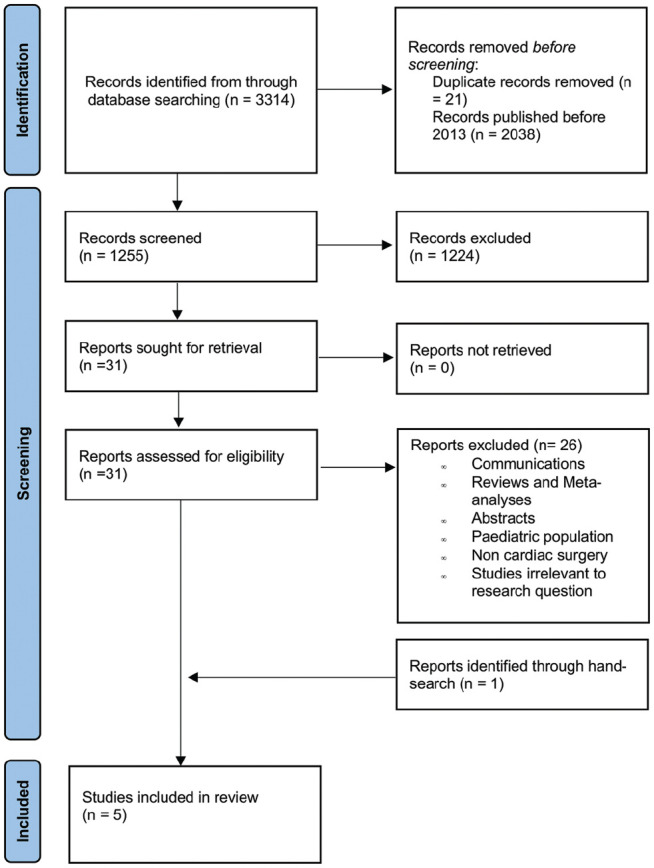
Preferred Reporting Items for Systematic Reviews and Meta-Analyses (PRISMA) flow diagram.

A total of 3,314 studies were retrieved from the systematic search, of which 4 met the criteria for inclusion in the final analysis. One study meeting the inclusion criteria was identified through hand search. Figure 1 shows the PRISMA flowchart for study selection. Included studies were published between 2017 and 2022; all studies were observational, single-center cohorts. There were 4 that originated from Germany and 1 from Sweden.

Table 1 shows the details of the included studies. Three studies were based on risk-adjusted populations. A total of 2,038 patients were included in the final analysis. The number of patients in each study ranged from 268 to 524.

**Table 1.** Summary of Included Studies.

Author	Year of publication	Country	Patients	Study design	Enrollment start and end dates	Population comparability	Selected outcomes
Saeed et al.^3^	2022	Germany	524 436 SC, 88 PC	Retrospective, single-center, nonrandomized	January 2018 April 2022	Propensity score matching	Any access site complication, in-hospital mortality, any wound complication, any vascular complication, lymphatic fistula, stroke, procedural duration, and hospital LOS
Sugimura et al.^4^	2022	Germany	311 218 SC, 93 PC	Retrospective, single-center, nonrandomized	September 2012 December 2020	No adjustment	Any access site complication, any wound complication, any vascular complication, lymphatic fistula, femoral/iliac stenosis, stroke, procedural duration, and hospital LOS
El-Sayed Ahmad et al.^5^	2022	Germany	490 222 SC, 268 PC	Retrospective, single-center, nonrandomized	November 2018 January 2021	No adjustment	Any access site complication, in-hospital mortality, any wound complication, any vascular complication, lymphatic fistula, stroke, procedural duration, and hospital LOS
Kastengren et al.^6^	2020	Sweden	268 147 SC, 121 PC	Prospective, single-center, nonrandomized	February 2016 December 2018	Propensity score matching	Any acess site complication, in-hospital mortality, any wound complication, any vascular complication, femoral/iliac stenosis, and hospital LOS
Moschovas et al.^2^	2017	Germany	445 92 SC, 353 PC	Retrospective, single-center, nonrandomized	October 2010 March 2015	Multivariable regression	Any access site complication, any wound complication, any vascular complication, lymphatic fistula, femoral/iliac stenosis, stroke, procedural duration, and hospital LOS

Abbreviations: LOS, length of stay; PC, percutaneous cannulation; SC, surgical cannulation.

### Patient Characteristics

Supplemental Table 4 summarizes the demographic data of the patient population in each study. The mean age ranged from 57.0 to 68.0 years, the percentage of female patients ranged from 19.3% to 55.5%, the mean body mass index ranged from 25.3 to 27.5 kg/m^2^, the percentage of hypertension ranged from 56.0% to 77.3%, the percentage of diabetes ranged from 1.8% to 23.9%, the percentage of peripheral vascular disease ranged from 0% to 15.8%, the percentage of atrial fibrillation ranged from 10.0% to 42.5%, and the percentage of prior cardiac surgery ranged from 0% to 9.6%.

The percentage of single-valve and double-valve surgery ranged from 55.4% to 95.3% and from 3.5% to 34.8%, respectively. The percentage of mitral, aortic, and tricuspid valve ranged from 47.0% to 99.5%, 0% to 42.8%, and 0% to 10.8%, respectively. One study displayed the percentage of elective for surgical versus percutaneous cannulation (94.1% vs 97.0%), urgent (4.5% vs 2.6%), and emergent surgery (1.4% vs 0.4%).^5^

### Meta-Analysis

Table 2 outlines the detailed results of the meta-analysis.

**Table 2.** Outcomes Summary.

Outcome	Number of studies	Number of patients	Effect estimate (95% CI)	*P* value
Any access site complication	5	1,722	OR 3.09 (1.87 to 5.10)	<0.01
Perioperative mortality	4	1,411	OR 1.45 (0.23 to 9.04)	0.66
Any wound complication	5	1,722	OR 10.10 (3.31 to 30.85)	<0.01
Any vascular complication	5	1,722	OR 0.75 (0.39 to 1.44)	0.22
Lymphatic complications	4	1,504	OR 9.37 (2.15 to 40.81)	0.01
Femoral/iliac stenosis	3	1,153	OR 0.52 (0.11 to 2.51)	0.41
Stroke	3	1,019	OR 0.47 (0.06 to 3.35)	0.45
Procedural duration	4	1,504	SMD 0.31 (0.12 to 0.51)	<0.01
Hospital length of stay	5	1,722	SMD 0.16 (–0.03 to 0.36)	0.10

Abbreviations: CI, confidence interval; OR, odds ratio; SMD, standardized mean difference.

#### Primary outcome

**Fig. 2.**
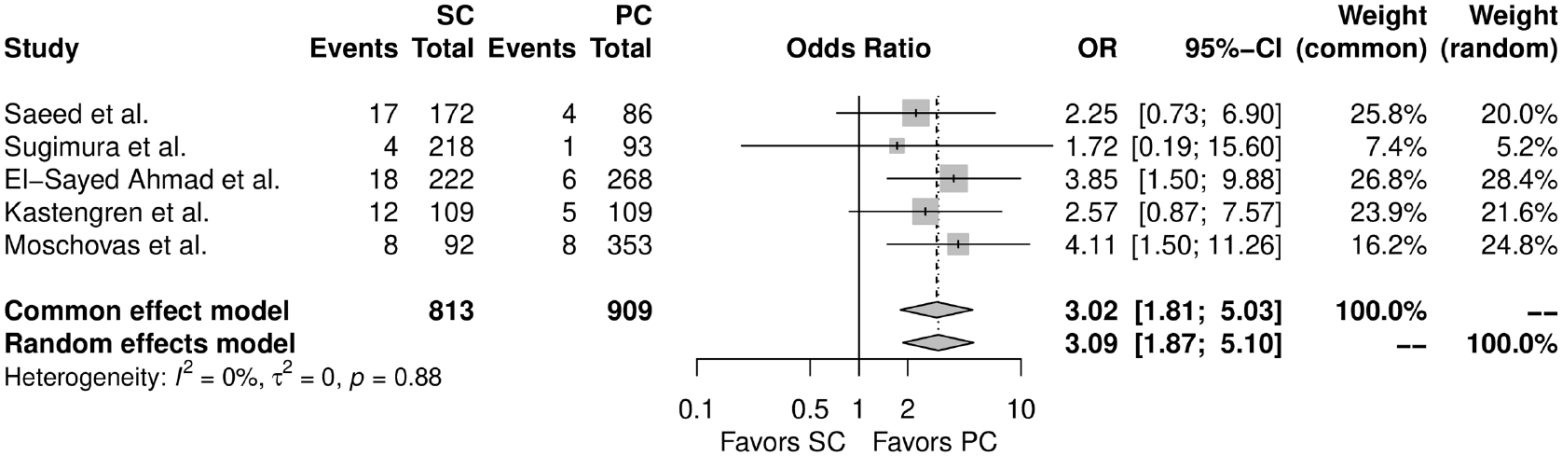
Forest plot for any access site complication. CI, confidence interval; OR, odds ratio; PC, percutaneous cannulation; SC, surgical cannulation.

Figure 2 shows the forest plot for any access site complication. When compared with percutaneous femoral cannulation, the patients who underwent surgical femoral cannulation showed a higher incidence of any access site complication (OR = 3.09, 95% CI: 1.87 to 5.10, *P* < 0.01). Supplemental Figure 1 shows the leave-one-out analysis indicating that most of the studies confirm the robustness of the analysis. Supplemental Figure 2 shows the funnel plot indicating no evidence of publication bias.

#### Secondary outcomes

**Fig. 3.**
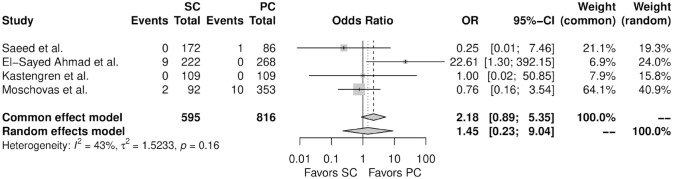
Forest plot for perioperative mortality. CI, confidence interval; OR, odds ratio; PC, percutaneous cannulation; SC, surgical cannulation.

**Fig. 4.**
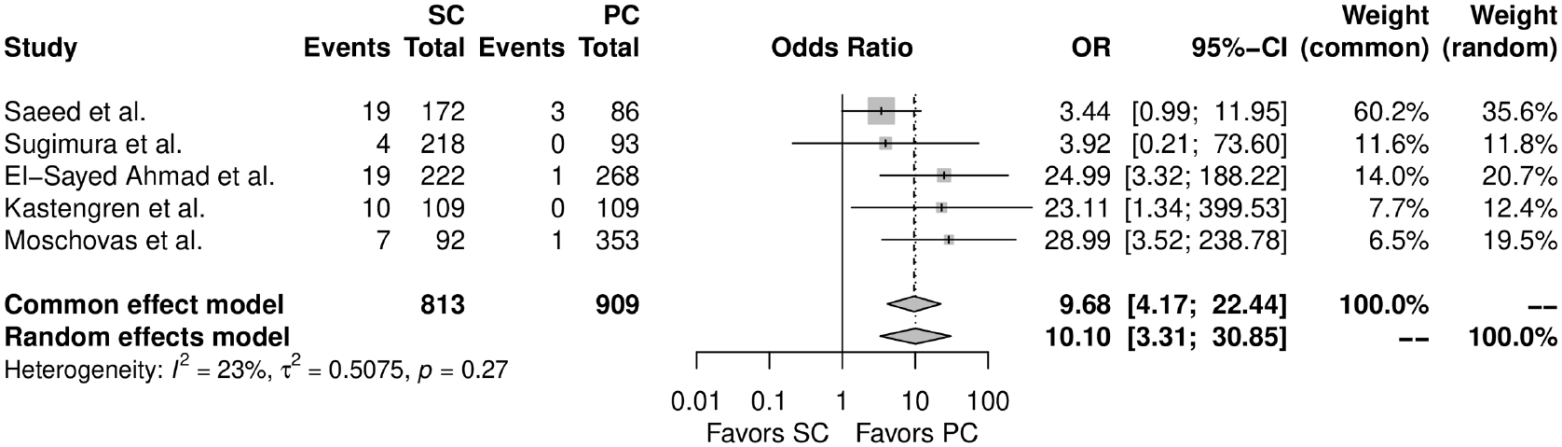
Forest plot for any wound complication. CI, confidence interval; OR, odds ratio; PC, percutaneous cannulation; SC, surgical cannulation.

**Fig. 5.**
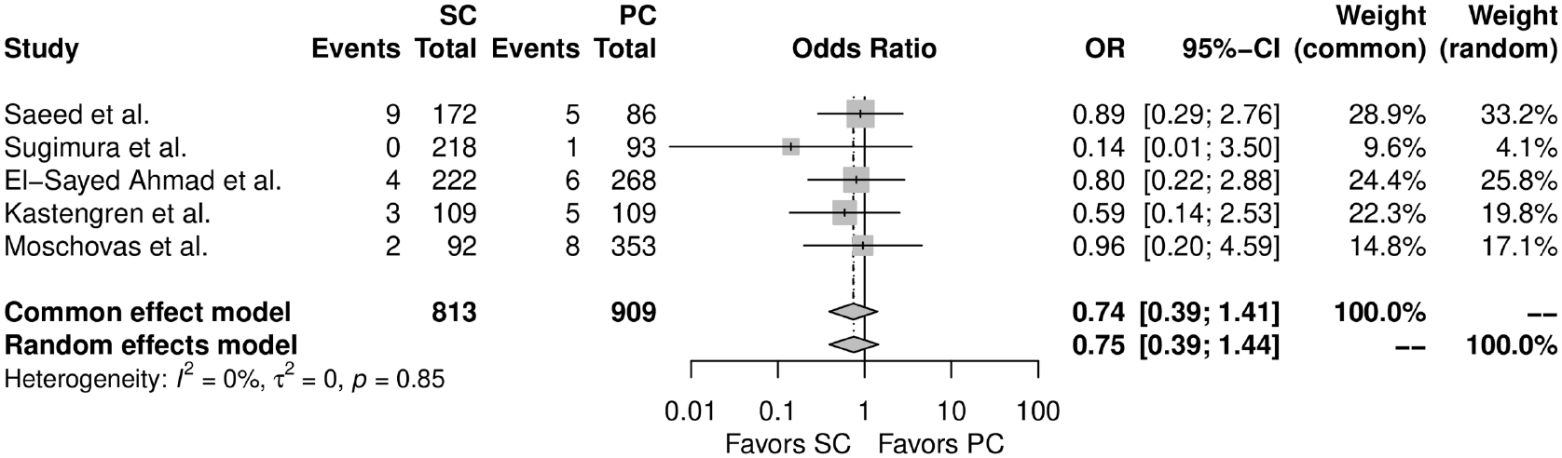
Forest plot for any vascular complication. CI, confidence interval; OR, odds ratio; PC, percutaneous cannulation; SC, surgical cannulation.

Figure 3 shows the forest plot for perioperative mortality. There was no significant difference between the 2 therapy groups (OR = 1.45, 95% CI: 0.23 to 9.04, *P* = 0.66). Figure 4 shows the forest plot for any wound complication. When compared with percutaneous femoral cannulation, the patients who underwent surgical femoral cannulation showed a higher incidence of any wound complication (OR = 10.10, 95% CI: 3.31 to 30.85, *P* < 0.01). Figure 5 shows the forest plot for any vascular complication. There was no significant difference between the 2 therapy groups (OR = 0.75, 95% CI: 0.39 to 1.44, *P* = 0.22).

Supplemental Figure 3 shows the forest plot for lymphatic complications. When compared with percutaneous femoral cannulation, the patients who underwent surgical femoral cannulation showed a higher incidence of lymphatic complications (OR = 9.37, 95% CI: 2.15 to 40.81, *P* < 0.01). Supplemental Figure 4 shows the forest plot for femoral/iliac stenosis. There was no significant difference between the 2 therapy groups (OR = 0.52, 95% CI: 0.11 to 2.51, *P* = 0.41). Supplemental Figure 5 shows the forest plot for stroke. There was no significant difference between the 2 therapy groups (OR = 0.47, 95% CI: 0.06 to 3.35, *P* = 0.45). Supplemental Figure 6 shows the forest plot for procedural duration. When compared with percutaneous femoral cannulation, the patients who underwent surgical femoral cannulation showed a longer procedural duration (SMD = 0.31, 95% CI: 0.12 to 0.51, *P* < 0.01). Supplemental Figure 7 shows the forest plot for hospital length of stay. There was no significant difference between the 2 therapy groups (SMD = 0.16, 95% CI: −0.03 to 0.36, *P* = 0.10).

## Discussion

Our analysis suggests that femoral cannulation through surgical incision in patients undergoing MICS is associated with a higher incidence of access site complications (especially wound complication and lymphatic complications) and with a longer procedure time when compared with surgery with percutaneous femoral cannulation.

Our results are relevant as an extensive number of patients receive MICS every year. In Germany in 2021, 55.7% of 6,052 mitral valve cases were performed using MICS.^7^ Similarly, a Society of Thoracic Surgeons database analysis showed that in the United States between 2014 and 2018, 10,238 (24.9%) mitral valve operations were performed minimally invasively, with a clear tendency toward increased MICS usage (19% in 2014 vs 30% in 2018).^8^ Importantly, the great majority of those procedures used femoral canulation.^8^ If we take into account the similar percentages of MICS surgery reported by a number of other countries all over the world (e.g., 30.8% MICS for mitral valve surgery in Japan,^9^ some centers in China,^10^ etc.), this would result in more than 100,000 MICS procedures performed annually with femoral cannulation.

Therefore, it may be surprising that despite the large numbers of MICS with groin cannulation worldwide, we could identify only 5 publications reporting results/comparisons for surgical and percutaneous groin cannulation. This fact is in sharp contrast with the field of invasive procedures/transcatheter aortic valve replacement, in which ACDs and percutaneous cannulation have become an established standard and a number of observational and even randomized studies have been conducted.^11–14^

An important aspect of our results is the strength of the observed associations. Patients with surgical cannulation were 12 times more likely to develop wound infections (OR = 12.62, *P* < 0.01) and 15 times more likely to develop a lymphatic complication, including lymphatic fistula and lymphocele, than those with percutaneous cannulation. This is especially relevant for clinical practice, as the incidence of groin complications after surgical cutdown have been reported to be more than 10%, and seromas account for most of the events,^15^ which would potentially translate to approximately more than 10,000 affected patients every year. Thus, our analysis provides for the first quantification of the benefits associated with percutaneous cannulation, which make these data especially valuable for improving patient outcomes or for serving as a base for guiding potential ACD reimbursement models in health systems around the world. This is especially relevant as groin complications may require lengthy and costly hospital stays.

Finally, our results show that both cannulation techniques, surgical and percutaneous, are safe and that using each of them does not compromise perioperative mortality. Thus, MICS remains a less-invasive, appealing, and, most importantly, safe surgical approach, irrespective of the cannulation technique used.

### Study Strengths and Limitations

This is the first meta-analysis to address this important topic. Moreover, we analyzed 8 different outcomes besides perioperative mortality. However, this work has the intrinsic limitations of observational series, including the risk of methodological heterogeneity of the included studies and residual confounders. Heterogeneity of the articles included the inconsistent reporting of the use of vascular ultrasound before discharge of patients who received percutaneous cannulation. One study did not report routine postoperative ultrasound of the peripheral vessels, and 2 studies performed vascular imaging on demand only. Only 2 centers performed routine postoperative ultrasound in the operation room or during the intensive care unit stay. Finally, no precise data regarding vascular complications were presented in terms of the specific rate of vascular perforations or retrograde dissections, which are serious complications that can result in dramatic postoperative scenarios.

## Conclusions

Our analysis suggests that the surgical cannulation technique in MICS is associated with a higher incidence of any access site complication (especially wound complication and lymphatic fistula) and a longer procedural time compared with the percutaneous cannulation. Importantly, there was no difference in perioperative mortality. Prospective, multicenter, randomized studies are needed in the future to validate the observed differences in postoperative outcomes as well as examine the potential cost differences between the 2 cannulation approaches.

## Supplemental Material

sj-pdf-1-inv-10.1177_15569845241241534 – Supplemental material for Percutaneous Versus Surgical Femoral Cannulation in Minimally Invasive Cardiac Surgery: A Systematic Review and Meta-Analysis Supplemental material, sj-pdf-1-inv-10.1177_15569845241241534 for Percutaneous Versus Surgical Femoral Cannulation in Minimally Invasive Cardiac Surgery: A Systematic Review and Meta-Analysis by Hristo Kirov, Tulio Caldonazo, Angelique Runkel, Johannes Fischer, Panagiotis Tasoudis, Murat Mukharyamov, Gianmarco Cancelli, Michele Dell’Aquila and Torsten Doenst in Innovations
