# Supplementary material for: Percutaneous Versus Surgical Femoral Cannulation in Minimally Invasive Cardiac Surgery: A Systematic Review and Meta-Analysis
Source: Innovations (Phila). 2024 Apr 11;19(3):247–53. doi: 10.1177/15569845241241534 (PMC11385621; doi:10.1177/15569845241241534)

Supplemental Table 1. Search Strategy for Ovid MEDLINE.

((("femor"[All Fields] OR "femorals"[All Fields] OR "femur"[MeSH Terms] OR "femur"[All Fields] OR "femoral"[All Fields]) AND ("cannulate"[All Fields] OR "cannulated"[All Fields] OR "cannulating"[All Fields] OR "cannulator"[All Fields] OR "cannulators"[All Fields] OR "cannulisation"[All Fields] OR "cannulization"[All Fields] OR "cannulized"[All Fields] OR "catheterization"[MeSH Terms] OR "catheterization"[All Fields] OR "cannulation"[All Fields] OR "cannulations"[All Fields])) OR (("groin"[MeSH Terms] OR "groin"[All Fields] OR "groins"[All Fields]) AND ("cannulate"[All Fields] OR "cannulated"[All Fields] OR "cannulating"[All Fields] OR "cannulator"[All Fields] OR "cannulators"[All Fields] OR "cannulisation"[All Fields] OR "cannulization"[All Fields] OR "cannulized"[All Fields] OR "catheterization"[MeSH Terms] OR "catheterization"[All Fields] OR "cannulation"[All Fields] OR "cannulations"[All Fields]))) AND ("surgical procedures, operative"[MeSH Terms] OR ("surgical"[All Fields] AND "procedures"[All Fields] AND "operative"[All Fields]) OR "operative surgical procedures"[All Fields] OR "surgical"[All Fields] OR "surgically"[All Fields] OR "surgicals"[All Fields]) AND ("percutaneous"[All Fields] OR "percutaneously"[All Fields] OR "percutanous"[All Fields]) AND ("thoracic surgery"[MeSH Terms] OR ("thoracic"[All Fields] AND "surgery"[All Fields]) OR "thoracic surgery"[All Fields] OR ("heart"[All Fields] AND "surgery"[All Fields]) OR "heart surgery"[All Fields] OR "cardiac surgical procedures"[MeSH Terms] OR ("cardiac"[All Fields] AND "surgical"[All Fields] AND "procedures"[All Fields]) OR "cardiac surgical procedures"[All Fields] OR ("heart"[All Fields] AND "surgery"[All Fields]))
**Translations**
femoral: "femor"[All Fields] OR "femorals"[All Fields] OR "femur"[MeSH Terms] OR "femur"[All Fields] OR "femoral"[All Fields]<br>cannulation: "cannulate"[All Fields] OR "cannulated"[All Fields] OR "cannulating"[All Fields] OR "cannulator"[All Fields] OR "cannulators"[All Fields] OR "cannulisation"[All Fields] OR "cannulization"[All Fields] OR "cannulized"[All Fields] OR "catheterization"[MeSH Terms] OR "catheterization"[All Fields] OR "cannulation"[All Fields] OR "cannulations"[All Fields]<br>groin: "groin"[MeSH Terms] OR "groin"[All Fields] OR "groins"[All Fields]<br>cannulation: "cannulate"[All Fields] OR "cannulated"[All Fields] OR "cannulating"[All Fields] OR "cannulator"[All Fields] OR "cannulators"[All Fields] OR "cannulisation"[All Fields] OR "cannulization"[All Fields] OR "cannulized"[All Fields] OR "catheterization"[MeSH Terms] OR "catheterization"[All Fields] OR "cannulation"[All Fields] OR "cannulations"[All Fields]<br>surgical: "surgical procedures, operative"[MeSH Terms] OR ("surgical"[All Fields] AND "procedures"[All Fields] AND "operative"[All Fields]) OR "operative surgical procedures"[All Fields] OR "surgical"[All Fields] OR "surgically"[All Fields] OR "surgicals"[All Fields]<br>percutaneous: "percutaneous"[All Fields] OR "percutaneously"[All Fields] OR "percutanous"[All Fields]<br>heart surgery: "thoracic surgery"[MeSH Terms] OR ("thoracic"[All Fields] AND "surgery"[All Fields]) OR "thoracic surgery"[All Fields] OR ("heart"[All Fields] AND "surgery"[All Fields]) OR "heart surgery"[All Fields] OR "cardiac surgical procedures"[MeSH Terms] OR ("cardiac"[All Fields] AND "surgical"[All Fields] AND "procedures"[All Fields]) OR "cardiac surgical procedures"[All Fields] OR ("heart"[All Fields] AND "surgery"[All Fields])

Supplemental Table 2. Newcastle-Ottawa Risk of Bias Assessment Scale.

Study	Selection	Comparability	Outcome/ exposure
Saeed	****	**	***
Sugimura	****	*	***
El-Sayed Ahmad	****	*	***
Kastengren	****	**	***
Moschovas	****	**	***

Supplemental Table 3. Surgical Techniques and Devices Used in the Individual Studies.

Study	Percutaneous arterial cannulation	Percutaneous venous cannulation	Surgical description
Saeed	Primary suture-based technique (Perclose ProGlide) complemented by small-sized plug-based closure device (6-F or 8 -F AngioSeal)	Single suture around venous line and manual compression	Surgical cut-down, arterial puncture under direct vision, purse string suture
Sugimura	Suture-based technique (Perclose ProGlide)	Suture based technique (Perclose ProGlide) and manual compression	Surgical cut-down, arterial puncture under direct vision, purse string suture
El-Sayed Ahmad	Ultrasound-guided MANTA VCD (14 and 18F)	Manual compression	Surgical cut-down, arterial puncture under direct vision, direct suture
Kastengren	MANTA VCD (18 F)	Manual compression	Surgical cut down, arterial puncture under direct vision, purse-string-closure
Moschovas	Suture-based technique (Perclose ProGlide)	Manual compression	Surgical cut down, arterial puncture under direct vision

Supplemental Table 4. Demographics of Included Patients From the Selected Studies (Part 1).

	Age, mean ± SD		Female, %		BMI, mean ± SD		HP, %		DM, %		PVD, %		Afib, %		Prior cardiac surgery, %	
**Study**	**SC**	**PC**	**SC**	**PC**	**SC**	**PC**	**SC**	**PC**	**SC**	**PC**	**SC**	**PC**	**SC**	**PC**	**SC**	**PC**
Saeed	57±12	59.67±11	27.0	33.0	25.3±3.7	25.6±3.8	58.0	56.0	10.0	10.0	2.0	3.5	10.0	10.0	0.6	0
Sugimura	64.7±12.1	63.6±13.8	55.5	54.8	26.6±5.1	25.9±4.6	72.5	60.2	10.6	8.6	3.2	5.4	NR	NR	NR	NR
El-Sayed Ahmad	67±12	68±11	39.6	38.8	26.5±4.5	26.2±3.7	64.0	58.2	11.3	9.3	15.8	14.2	16.2	14.9	NR	NR
Kastengren	60.5±13	61.3±11	19.3	22.0	NR	NR	NR	NR	3.7	1.8	0	0	31.2	30.3	NR	NR
Moschovas	65±12	66±12	44.6	45.0	27.5±5.2	27±5.2	73.9	77.3	23.9	20.4	5.4	8.8	38.0	42.5	3.3	9.6

Abbreviations: Afib, atrial fibrillation; BMI, body mass index; DM, diabetes; HP, hypertension; NR, not reported; PC, percutaneous cannulation; PVD, peripheral vascular disease; SC, surgical cannulation; SD, standard deviation.

Supplemental Table 4. Demographics of Included Patients From the Selected Studies (Part 2).

	Elective surgery, %		Urgent surgery, %		Emergency, %		Single valve surgery, %		Double valve surgery, %		Mitral valve, %		Tricuspid valve, %		Aortic valve, %	
**Study**	**SC**	**PC**	**SC**	**PC**	**SC**	**PC**	**SC**	**PC**	**SC**	**PC**	**SC**	**PC**	**SC**	**PC**	**SC**	**PC**
Saeed	NR	NR	NR	NR	NR	NR	90.7	95.3	5.8	3.5	89.0	91.9	1.7	3.5	0	0
Sugimura	NR	NR	NR	NR	NR	NR	NR	NR	NR	NR	99.5	97.8	NR	NR	NR	NR
El-Sayed Ahmad	94.1	97.0	4.5	2.6	1.4	0.4	93.2	94.8	6.8	4.9	50.5	52.2	0	0.4	42.8	42.2
Kastengren	NR	NR	NR	NR	NR	NR	NR	NR	NR	NR	NR	NR	NR	NR	NR	NR
Moschovas	NR	NR	NR	NR	NR	NR	55.4	69.7	34.8	26.6	47.8	47.0	7.6	10.8	0	11.9

Abbreviations: NR, not reported; PC, percutaneous cannulation; SC, surgical cannulation.

Supplemental Fig. 1. Leave-one-out analysis for the primary endpoint (any access site complication).

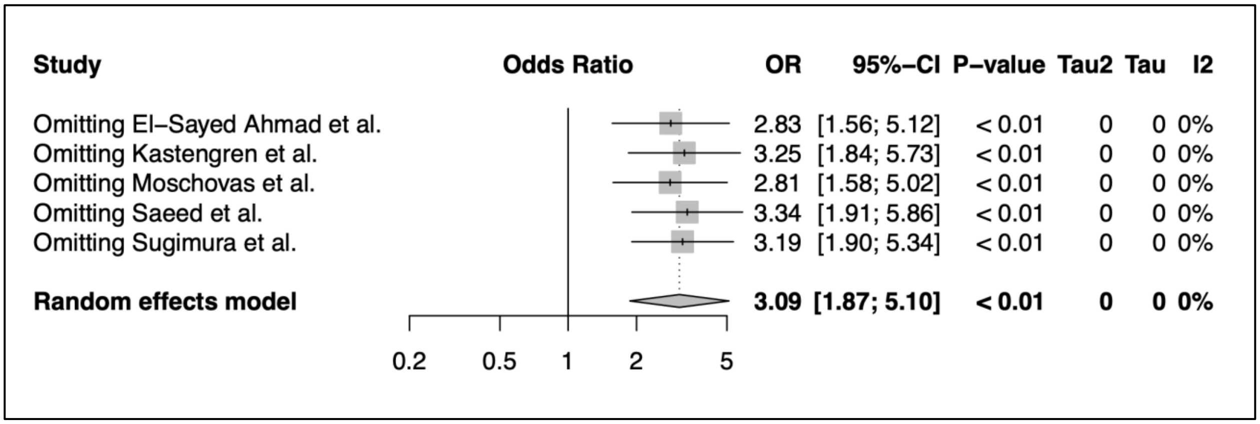

Supplemental Fig. 2. Funnel plot for the primary endpoint (any access site complication).

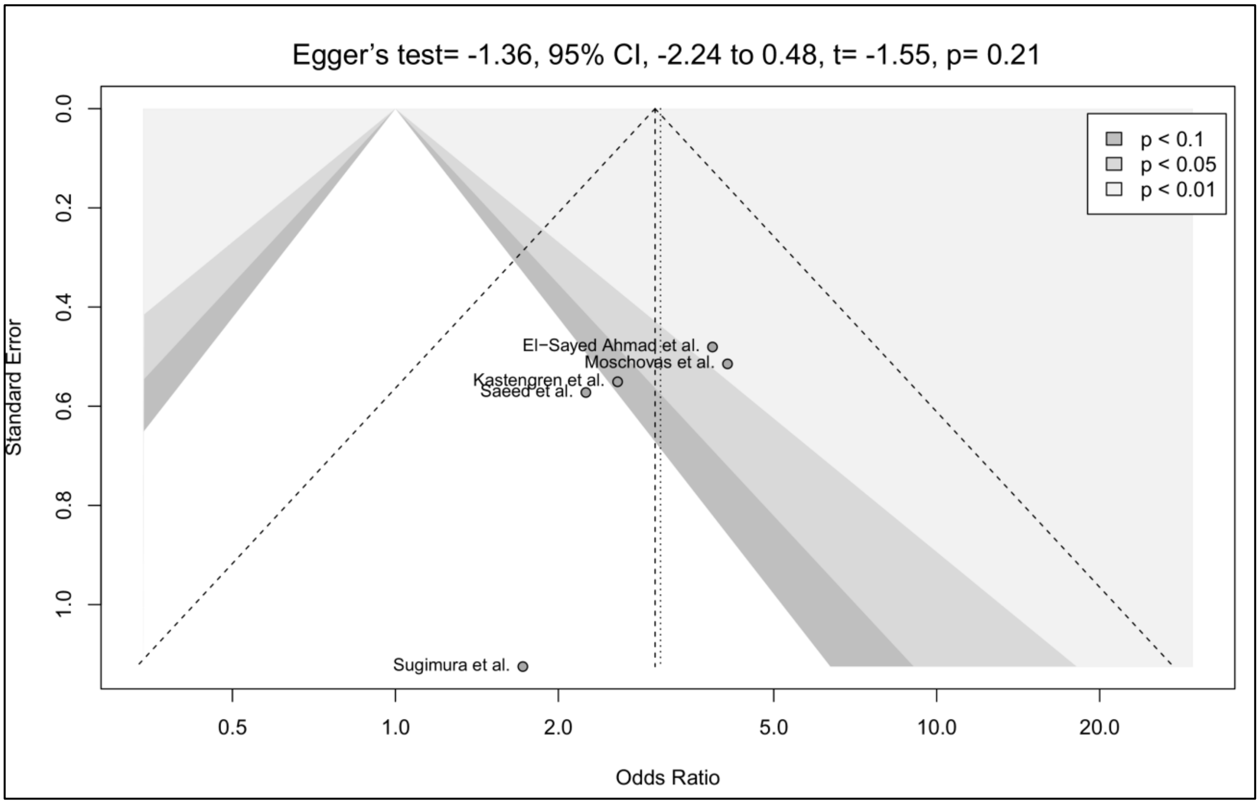

Supplemental Fig. 3. Forest plot for lymphatic complications.

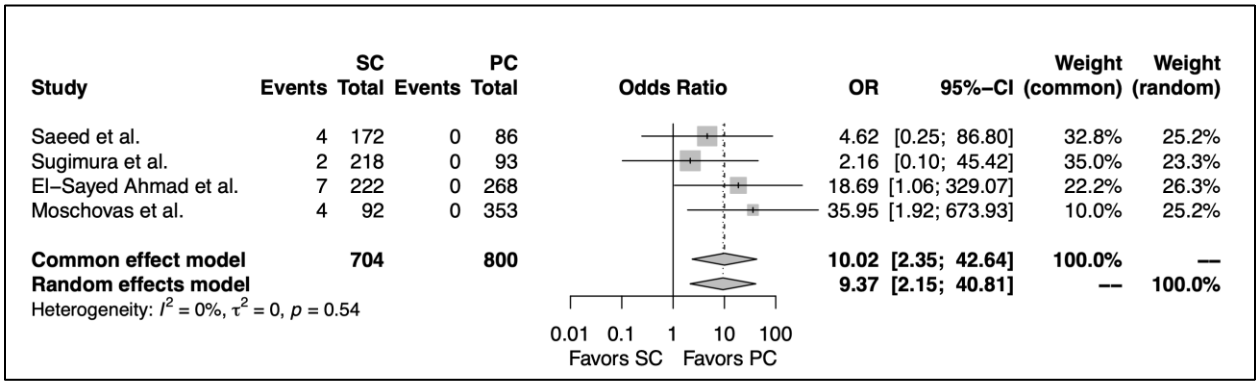

Supplemental Fig. 4. Forest plot for femoral/iliac stenosis.

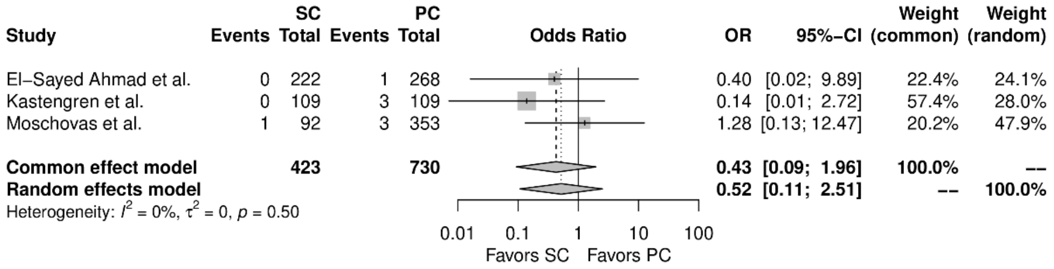

Supplemental Fig. 5. Forest plot for stroke.

Study	SC Events	SC Total	PC Events	PC Total	OR	95%–CI	Weight (common)	Weight (random)
Sugimura et al.	0	218	2	93	0.08	[0.00; 1.76]	31.1%	24.6%
El–Sayed Ahmad et al.	8	222	6	268	1.63	[0.56; 4.78]	46.7%	50.8%
Kastengren et al.	0	109	2	109	0.20	[0.01; 4.14]	22.2%	24.6%
**Common effect model**		**549**		**470**	**0.83**	**[0.35; 1.98]**	**100.0%**	**--**
**Random effects model**					**0.47**	**[0.06; 3.35]**	**--**	**100.0%**

Heterogeneity: $I^2 = 55\%$, $\tau^2 = 1.6935$, $p = 0.11$

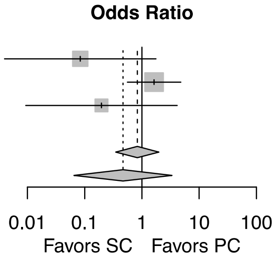

Supplemental Fig. 6. Forest plot for procedural duration.

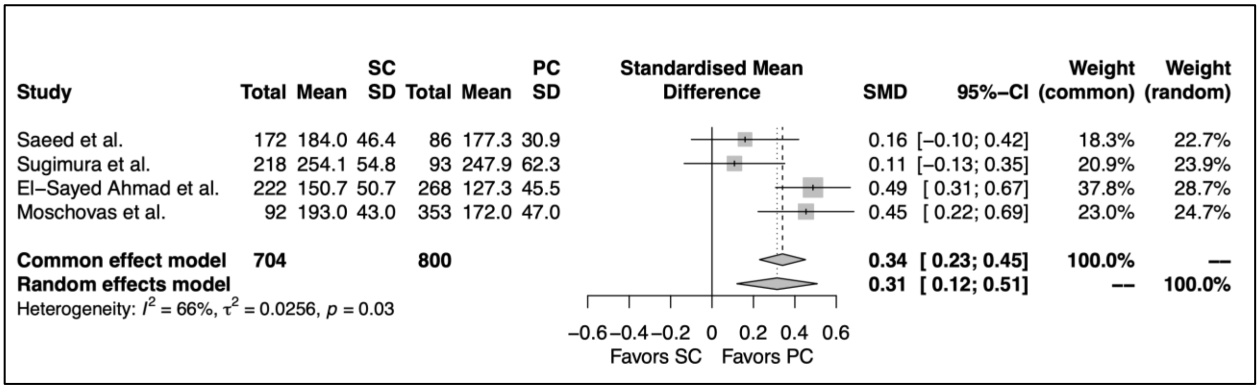

Supplemental Fig. 7. Forest plot for length of hospital stay.

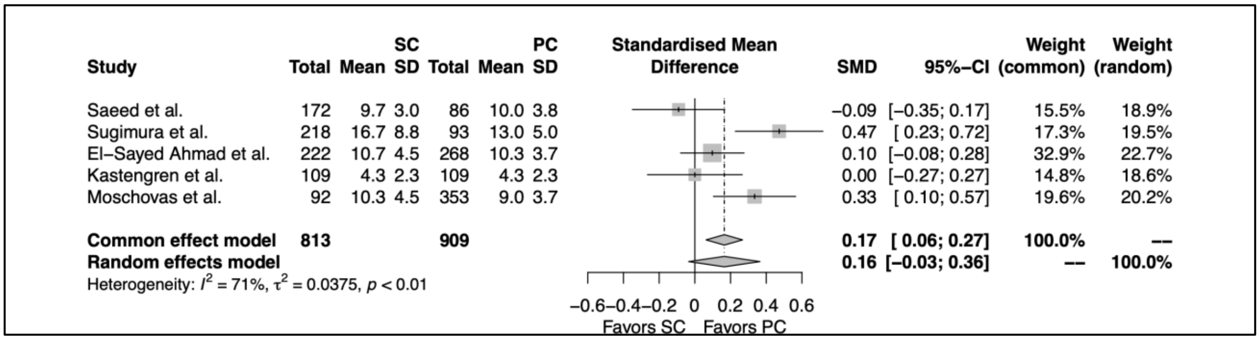

Supplement: sj-pdf-1-inv-10.1177_15569845241241534 – Supplemental material for Percutaneous Versus Surgical Femoral Cannulation in Minimally Invasive Cardiac Surgery: A Systematic Review and Meta-Analysis [file sj-pdf-1-inv-10.1177_15569845241241534.pdf]
